# Shenqi Shiyiwei Granule for breast cancer related fatigue-centered symptom cluster: protocol of a multicenter randomized double-blind placebo-controlled trial

**DOI:** 10.3389/fonc.2026.1941699

**Published:** 2026-10-19

**Authors:** Si-Yuan Li, Tong-Tong Wu, Yu-Xuan Zhang, Jia Zhao, Jing Hu, Gan-Lin Zhang, Guo-Wang Yang

**Affiliations:** 1 Department of Oncology, Beijing Hospital of Traditional Chinese Medicine, Capital Medical University, Beijing, China; 2 Beijing University of Chinese Medicine, Beijing, China; 3 Shandong Second Medical University, Weifang, Shandong, China; 4 Beijing Institute of Chinese Medicine, Beijing, China

**Keywords:** breast cancer, fatigue, oncology, symptom cluster, TCM

## Abstract

**Background:**

Breast cancer survivors frequently experience fatigue-centered symptom clusters—concurrent, interrelated symptoms including fatigue, pain, and dyspnea—that significantly impair quality of life and functional status. Despite the high prevalence of these symptoms, few interventional trials have targeted symptom clusters as a unified treatment endpoint. Current management relies predominantly on behavioral interventions with inconsistent efficacy, and no pharmacological agent has demonstrated high-quality evidence for cluster-level symptom control in this population.

**Methods:**

This multicenter, randomized, double-blind, placebo-controlled trial will enroll 262 postoperative breast cancer survivors (stage I–IIIA) with moderate-to-severe fatigue-centered symptom clusters from 11 hospitals across China. Eligible participants must have completed adjuvant therapy, exhibit an ECOG performance status of 0–1, and present with a MDASI-C fatigue-dyspnea-pain subscale score ≥4. Participants will be randomized 1:1 to receive either Shenqi Shiyiwei Granule or matching placebo for 60 days, with standardized psycho-exercise-dietary guidance provided to all participants throughout a 120-day study period. The primary endpoint is the change from baseline in the fatigue-dyspnea-pain symptom cluster score at 2 months, assessed using the MD Anderson Symptom Inventory-Chinese version (MDASI-C). Secondary endpoints include quality-of-life measures (MFSI-SF, FACT-B, TCM Spleen Qi Deficiency Scale) and cardiopulmonary functional capacity. Exploratory biomarker analyses will evaluate hypothalamic-pituitary-adrenal axis hormones, cytokine profiles, and gonadal hormone concentrations to elucidate potential mechanisms. Efficacy will be analyzed using mixed-effects models for repeated measures (MMRM) with prespecified sensitivity analyses for missing data assumptions.

**Discussion:**

This trial represents one of the first rigorous investigations targeting the fatigue-centered symptom cluster as a unified therapeutic endpoint in breast cancer survivors. By combining traditional Chinese herbal medicine with guideline-endorsed behavioral interventions, this study aims to provide high-quality evidence for a novel multi-target treatment strategy. The exploratory biomarker assessments may illuminate the biological mechanisms underlying symptom cluster interrelationships, establishing a foundation for future mechanistic and interventional research in cancer survivorship care.

**Clinical trial registration:**

[https://itmctr.ccebtcm.org.cn/mgt/project/user/user-project-view/0A267E22-1E0A-4A95-99B0-E5964124F203], The International Traditional Medicine Clinical Trial Registry [ITMCTR2025001511].

## 1 Introduction

Breast cancer is the leading cause of cancer death among women globally (1–3). With contemporary screening and multimodal treatment, 5-year relative survival now ranges from 86% to 99% for stage I–III disease in high-income settings (4, 5), shifting clinical priorities beyond tumor control toward long-term function and health-related quality of life (HRQoL) (6, 7).

Yet survivorship carries a persistent burden. Up to 80-90% of patients who receive surgery and adjuvant therapy suffer from fatigue (8–11). Pain, insomnia, anxiety, and dyspnea frequently co-occur with fatigue, potentially reflecting shared inflammatory and neuroendocrine mechanisms, with cardiopulmonary impairment and physical deconditioning additionally contributing to fatigue and dyspnea (12–17). Dodd et al. introduced the construct of symptom clusters—three or more concurrent, interrelated symptoms that may share underlying mechanisms—in 2001 (18). The fatigue-centered cluster is among the most prevalent in breast cancer survivors (19–21), yet few trials have tested cluster-targeted interventions (22). Current management relies largely on non-pharmacological strategies—structured exercise, sleep hygiene, lifestyle modification, whose efficacy for multi-symptom relief remains inconsistent across small, heterogeneous studies (23, 24). Pharmacological options include methylphenidate and short-term corticosteroids in advanced disease, but inconsistent efficacy and adverse effects, such as insomnia, anxiety, palpitations, hyperglycemia, and immunosuppression, limit their routine use, particularly in cancer survivors (25).

Differences in symptom selection, assessment instruments, and analytical methods may related to the variation in the reported composition of symptom clusters. The present trial focuses specifically on fatigue, dyspnea, and pain. In a 10-year follow-up cohort of 292 breast cancer survivors, sleep problems, fatigue, pain, and dyspnea were the most prevalent symptoms (26). Moreover, a network analysis of 1,330 survivors of seven cancer types, including 190 breast cancer survivors, identified fatigue as the most central symptom and demonstrated consistent direct relationships between fatigue and both pain and dyspnea across cancer types (27). These empirical findings, together with recurrent clinical observations of shortness of breath and pain among breast cancer survivors with clinically relevant fatigue at the participating centers, informed the prespecified selection of fatigue, dyspnea, and pain as the target symptom cluster. In this trial, dyspnea is operationalized using the MDASI-C item “shortness of breath.”

Shenqi Shiyiwei Granule is a traditional Chinese herbal formula, that has been investigated in different cancer-related settings. Network-pharmacology and molecular-docking analyses have identified potential immune- and apoptosis-related pathways in gastric cancer (28). In patients with stage II–III breast cancer receiving chemotherapy, the related Shiyiwei Shenqi tablet formulation was associated with changes in glucose, fatty-acid, bile-acid, and amino-acid metabolism (29). More broadly, traditional Chinese medicine interventions have shown potential benefits for cancer-related fatigue (30), and the related tablet formulation has also been evaluated in patients with cancer-related fatigue in combination with deep hyperthermia (31). Chinese expert consensus has further discussed the clinical application of Chinese patent medicines in cancer palliative care (32). These findings provide a preliminary basis for further evaluation of Shenqi Shiyiwei Granule in the management of multiple co-occurring symptoms. However, most previous studies involved patients receiving active anticancer treatment, used related tablet formulations or combination regimens, and focused on individual symptoms, hematological toxicity, immune parameters, or general functional status rather than a prespecified symptom cluster. The present trial extends this evidence by evaluating Shenqi Shiyiwei Granule in breast cancer survivors using a prespecified fatigue–dyspnea–pain symptom-cluster outcome. Integrating this agent with NCCN-endorsed psycho-exercise-dietary guidance (24, 33) could, in principle, address multiple symptom domains simultaneously. We therefore designed a randomized controlled trial to test whether this combined regimen reduces the fatigue-centered symptom cluster in postoperative breast cancer patients, compared with psycho-exercise-dietary guidance alone.

## 2 Methods and analysis

### 2.1 Design and setting

The Shenqi Shiyiwei Granule for breast cancer related fatigue-centered symptom cluster study is a randomized, multicenter, double-blind, placebo-controlled clinical trial to assess Shenqi Shiyiwei Granule in the management of fatigue-centered symptom cluster among breast cancer survivors, with the primary objective to evaluate the efficacy and safety of this herbal medicine (see **Figure 1** for the CONSORT flow diagram).

**Figure 1:**
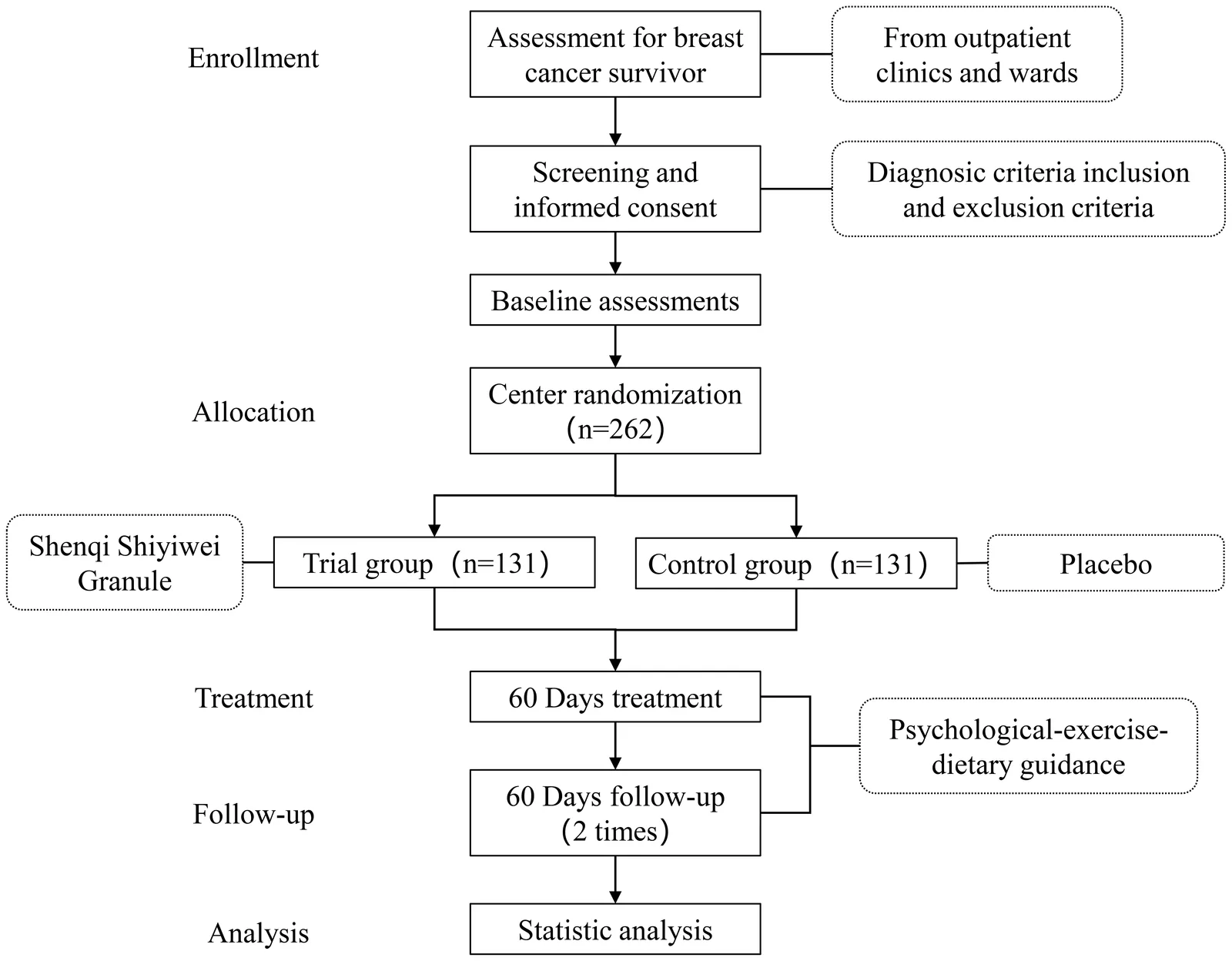
Flow diagram of the study.

A total of 262 postoperative breast cancer survivors (stage I-IIIA) who had completed adjuvant therapy will be enrolled in this study. Eligible patients will be randomized in a 1:1 allocation ratio to the active treatment group or the control group, with standardized psychological-exercise-dietary guidance provided to all participants regardless of group assignment. The trial protocol comprises a 60-day intervention period and a 60-day follow-up observation period (**Figure 2**). It is anticipated that 11 hospitals across China will participate in the present study.

**Figure 2:**
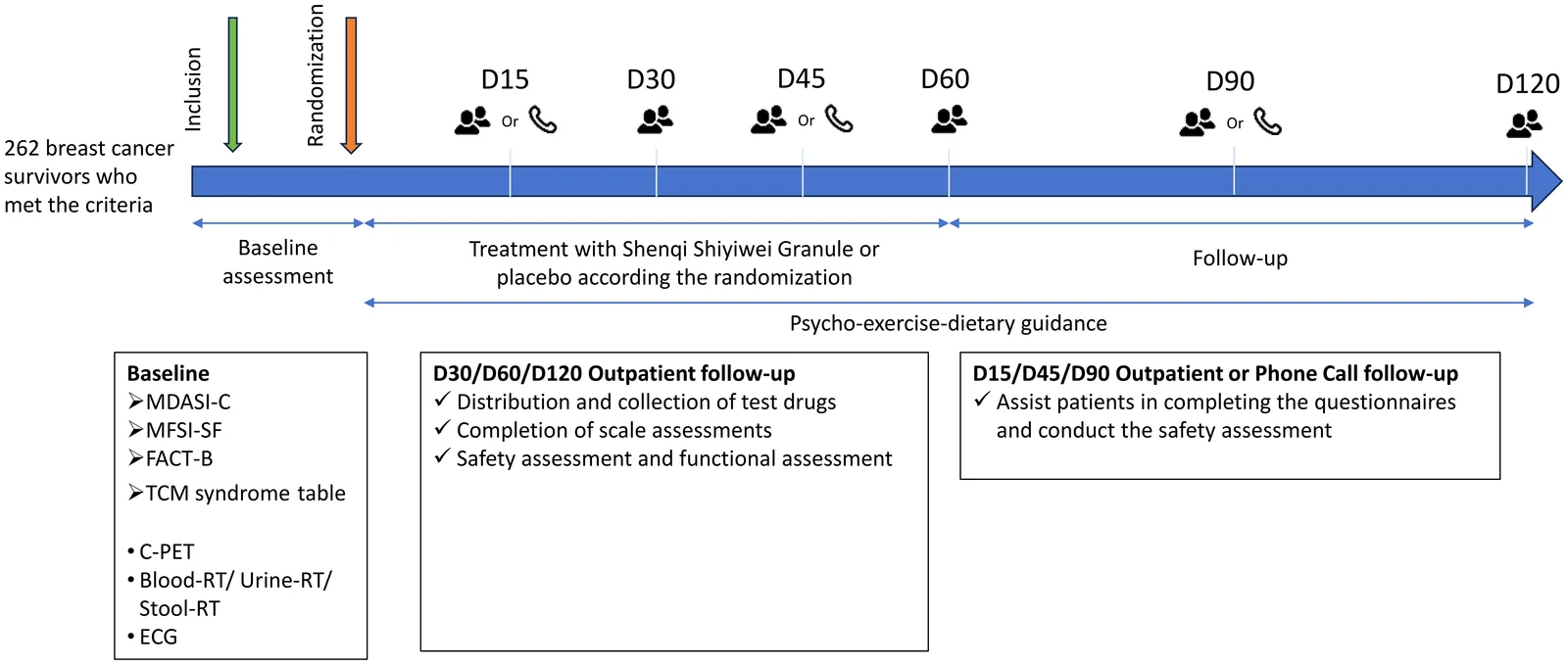
Data collection time.

### 2.2 Participants

#### 2.2.1 Enrollment

Participants will be recruited through multiple approaches, including poster postings at the institution, the use of digital platforms, and physician referrals. Contact numbers of the research team members will be listed in the recruitment advertisements to facilitate inquiries from potential participants. Enrollment will be carried out at the outpatient clinics and inpatient wards of 11 collaborating hospitals. Research candidates who meet the predefined inclusion and exclusion criteria and provide written informed consent will be enrolled in the trial.

#### 2.2.2 Inclusion criteria

(1) Female, aged 18 to 75 years old; (2) Patients with stage I to IIIA hormone receptor-negative breast cancer with a clear pathological diagnosis, who have undergone surgery and completed at least 3 months of adjuvant therapy or adjuvant intensified therapy, and have no signs of recurrence or metastasis; (3) ECOG-PS score of 0 to 1; (4) Moderate to severe fatigue-centered symptom cluster, that is, the MDASI-C fatigue, shortness of breath and pain score all ≥1, with their mean score ≥4; (5) Have not received treatment for fatigue-centered symptom cluster before enrollment; (6) No contraindications to exercise; (7) Have a certain ability to understand and express, voluntarily participate in this study, and sign the informed consent form; (8) According to the approved indications of Shenqi Shisanwei Granules, eligible patients should also meet the criteria of two main symptoms in the symptom diagnosis scale, or one main symptom combined with two secondary symptoms, in order to support the diagnosis of weakness (**Table 1** Symptom Rating Scale).

**Table 1:** Symptom rating scale.

Symptom rating scale					
Category	Symptom	None (0)	Mild (2)	Moderate (4)	Severe (6)
Primary Symptoms	Poor appetite/anorexia	0	2	4	6
	Physical fatigue and weakness	0	2	4	6
	Abdominal distension after eating	0	2	4	6
	Abnormal bowel movements	0	2	4	6
Secondary Symptoms	Mental fatigue and reluctance to speak	0	2	4	6
	Bland taste in the mouth without thirst	0	2	4	6
	Persistent dull abdominal pain	0	2	4	6
	Nausea and vomiting	0	2	4	6
	Epigastric stuffiness	0	2	4	6
	Borborygmus (bowel rumbling)	0	2	4	6
	Sallow yellow complexion	0	2	4	6
	Edema	0	2	4	6
	Difficulty passing stool (weak defecation)	0	2	4	6
	Pale tongue, white tongue and weak pulse				

#### 2.2.3 Exclusion criteria

(1) Patients with uncontrollable mental illness history and those with moderate to severe cognitive impairment; (2) Individuals who have experienced allergic reactions to traditional Chinese medicine or have been confirmed to be allergic to the ingredients of Shenqi Shiyiwei Granule; (3) Those expected to have disease progression of invasive breast cancer during the study period, who need radiotherapy treatment; (4) Those with other malignant tumors or those with severe heart, liver, or kidney dysfunction; (5) Those with severe comorbidities, active infections, severe bleeding tendencies, or abnormal hematopoietic function; (6) Those with active hepatitis B virus infection, active hepatitis C virus infection, or human immunodeficiency virus (HIV) infection history, or those with other acquired or congenital immune deficiency diseases; (7) Pregnant women and lactating women, or women of childbearing age who are unwilling to take effective contraceptive measures; (8) Participants who are currently involved in other clinical trials.

#### 2.2.4 Withdrawal criteria

(1) Tumor recurrence or progression; (2) Occurrence of inflammatory adverse events; (3) Safety concerns identified by the investigator; (4) Early termination or suspension of the clinical trial; (5) Participant withdrawal from the study for any reason.

#### 2.2.5 Sample size calculation

Preliminary small-sample observations by our research team demonstrated that among 30 patients with malignant tumors, the control group without any intervention presented a mean difference of -3.04 ± 2.28 in the MDASI symptom cluster score from baseline at 2 months post-enrollment.

Given that the minimum clinically important difference (MCID) of the MDASI-C scale has not been reported, it was hypothesized that the investigational drug could increase the absolute value of the 2-month mean difference by 1 point in patients with this specific symptom cluster (effect size [ES]≈0.44), which would indicate that the investigational drug meets clinical guideline requirements and holds clinical application value. Sample size calculation was conducted using the Two-Sample T-Tests with unequal variance model, with a two-sided alpha risk of 5% and power of 90%. Assuming 15% dropouts, 262 participants should be recruited.

### 2.3 Randomization, allocation and blinding

Block randomization will be adopted in this study. A single overall randomization sequence will be generated by an independent external statistician using a 1:1 allocation ratio. Randomly permuted blocks of four will be used for the first 260 participants, followed by a final balanced block of two, resulting in 131 participants per group. Randomization will not be stratified by study center or other prognostic factors, and the 11 participating centers will recruit competitively until the prespecified sample size is reached. The sequence will be implemented through a central interactive web response system (IWRS), configured and maintained by an independent system provider.

A double-blind method will be implemented throughout the trial. Trial drug will be labeled with unique drug codes containing randomization information, without displaying the treatment assignment. After screening and eligibility confirmation, the investigator will register the participant in the IWRS, which will automatically issue the corresponding study-drug code without revealing the treatment assignment. The investigator will then dispense the identically appearing study medication corresponding to that code.

The blind will be strictly maintained for both investigators and participants until the completion of the study and formal unblinding. Upon study conclusion, participants will be asked to guess their assigned group via a questionnaire, which will be used to verify whether the blinding was successfully maintained throughout the trial.

### 2.4 Interventions

Participants will receive Shenqi Shiyiwei Granule or placebo from day 0 to day 60, and all of them will receive psycho-exercise-dietary guidance throughout 120 days study period equally.

#### 2.4.1 Trial drug

Shenqi Shiyiwei Granule (NMPA Approval Number: Z10980002) and its matching placebo granule were uniformly supplied by Jiangxi Shangao Pharmaceutical Co., Ltd. Shenqi Shiyiwei Granule containing 11 herbal ingredients. Its verified composition, excipients, unit strength (2 g/sachet, equivalent to 5 g of herbal materials), regulatory information, and administration are detailed in **Supplementary Table S1**. The placebo consisted primarily of 5% Shenqi Shiyiwei Granule, dextrin, and starch, formulated to ensure strict similarity in appearance, odor, texture, and taste between the active and placebo products.

#### 2.4.2 Psycho-exercise-dietary guidance

In accordance with the 2025 NCCN Clinical Practice Guidelines in Oncology for Cancer-Related Fatigue (CRF), patients will receive integrated psycho-exercise-dietary guidance. These interventions encompass emotional distress management, and promotion of adaptive coping strategies; provision of health education covering disease- and treatment-related knowledge, non-pharmacological interventions, and coping skills; delivery of dietary guidance and adjustment recommendations for patients and their family members; and encouragement to engage in moderate-intensity physical activity (e.g., yoga) during and following anticancer treatment.

#### 2.4.3 Concomitant care

All eligible participants will have completed mandatory postoperative pharmacotherapy for breast cancer prior to enrollment, thereby precluding the concurrent use of antineoplastic agents during the study. Concomitant medications required for managing preexisting comorbidities (e.g., hypertension, coronary heart disease, diabetes mellitus, or sleep disturbance) are permitted, provided that their generic names, dosages, frequencies, and durations are fully documented for subsequent analysis and final reporting.

### 2.5 Outcomes

Scale assessments will be completed, and required laboratory tests as well as clinical examinations will be conducted for participants at specified time points throughout the study (**Table 2** Study schedule).

**Table 2:** Study schedule.

Timepoint	Study period							
	Enrolment	Allocation	Treatment				Follow-up	
	-7 to 0 days	0	D15	D30	D45	D60	D90	D120
Enrollment								
Medical records	×							
Eligibility screen	×							
Informed consent	×							
Allocation		×						
Interventions								
SQ granule								
Placebo granule								
P-E-D guidance								
Assessments								
MDASI-C		×	×	×	×	×	×	×
MFSI-SF		×	×	×	×	×	×	×
FACT-B		×	×	×	×	×	×	×
TCM Syndrome Scale		×	×	×	×	×	×	×
CPET		×				×		×
Exploratory Endpoints		×				×		×
Safety evaluation								
Assay		×				×		×
Adverse events		×	×	×	×	×	×	×
Trial drug								
Drug Dispensing		×		×				
Drug Retrieval				×		×		

SQ granule, Shenqi Shiyiwei granule; P-E-D guidance, psycho-exercise-dietary guidance; CPET, cardiopulmonary exercise testing.

#### 2.5.1 Primary efficacy analysis

Difference from baseline in the fatigue-dyspnea-pain symptom cluster score: Using the MDASI-C scale, assessments will be performed every 15 days throughout the treatment period. The symptom-cluster score is calculated as the arithmetic mean of the fatigue, shortness-of-breath, and pain item scores. The difference from baseline in the target symptom cluster score at 2 months post-enrollment is defined as the primary outcome.

#### 2.5.2 Secondary efficacy analysis

Secondary endpoints fall into two domains. For quality of life, patient-reported outcomes will be assessed every 15 days during the 60 days treatment phase using the MD Anderson Symptom Inventory–Chinese (MDASI-C), the Multidimensional Fatigue Symptom Inventory–Short Form (MFSI-SF), the Functional Assessment of Cancer Therapy–Breast (FACT-B), and a TCM-based Spleen Qi Deficiency symptom scale; change from baseline at D60 constitutes the prespecified quality-of-life endpoint. For functional status, cardiopulmonary exercise testing (CPET) will be performed at baseline, D60, and D120, with change in peak oxygen uptake (VO_2_peak) at D60 as the functional endpoint.

#### 2.5.3 Safety observations and exploratory endpoints

For safety evaluation, participants will undergo vital sign monitoring and laboratory assessments including complete blood count, urinalysis, stool routine, liver and kidney function tests, tumor marker assays, and ECG at baseline, at intervals during the treatment phase, and at scheduled time points throughout the follow-up period.

Additional blood samples will be collected from participants at designated time points for exploratory analyses. These exploratory assays will include assessments of the hypothalamic-pituitary-adrenal (HPA) axis, cytokine profiling, and gonadal hormone concentrations measurements, to preliminarily elucidate the potential biological mechanisms underlying fatigue-centered symptom cluster.

### 2.6 Data

#### 2.6.1 Data collection

Data will be entered into the electronic Case Report Form (e-CRF) as follows: participants will be assisted by investigators in completing entries via the Electronic Patient Reported Outcomes (ePRO) system. In addition, for participants who have difficulty operating smartphones, investigators may alternatively input follow-up data via the Clinical Research Coordinator (CRC) portal. Given that all our data collection processes are electronic, the system automatically verifies data rationality during entry, and e-CRF could facilitating information traceability. We encourage patients to attend face-to-face follow-up to achieve optimal outcomes, while telephone follow-up is also arranged at specific time points for optional access. To ensure data quality, double data entry will be employed for primary outcome measures, and automated range checks, logic validations, and consistency queries will be programmed to identify implausible or missing values in real time.

#### 2.6.2 Statistical analysis populations

Three analysis populations were prespecified. The intention-to-treat (ITT) population—serving as the primary analysis set for all efficacy endpoints—included all randomized participants analyzed according to assigned treatment, irrespective of protocol adherence or study completion. For sensitivity analyses, a per-protocol (PP) population was defined, restricted to participants completing the study without major deviations (e.g., prohibited concomitant therapy, ≥20% missed sessions, or unblinding). Safety analyses encompassed all participants who received at least one treatment session and underwent at least one post-baseline assessment.

#### 2.6.3 Efficacy analysis

Primary Efficacy Analysis: Changes in symptom cluster scores were evaluated using a mixed-effects model for repeated measures (MMRM). Fixed effects included treatment group, visit (categorical), their interaction, and baseline score; participant was modeled as a random effect. Within-subject correlations across biweekly assessments were accommodated via an unstructured covariance matrix—selected based on Akaike Information Criterion among candidate structures. The prespecified primary contrast was the between-group difference at Month 2, reported with 95% CI; two-sided α was set at 0.05.

Cardiopulmonary Exercise Testing: For CPET outcomes (VO_2_peak, anaerobic threshold, exercise duration), a constrained longitudinal data analysis (cLDA) model assessed trajectories across three timepoints: baseline, end-of-treatment, and end-of-follow-up. This approach constrains baseline means to equality across arms, improving efficiency when randomization ensures comparability. The Month-2 functional endpoint will be analyzed by ANCOVA, adjusting for baseline CPET value, age, and sex. Model assumptions (normality of residuals, homoscedasticity) were verified graphically; non-normal distributions prompted sensitivity analysis via bootstrap percentile CIs.

#### 2.6.4 Handling of missing data

The MMRM framework accommodates incomplete data under a missing-at-random (MAR) assumption, leveraging all observed values without requiring imputation. To probe sensitivity to this assumption, three complementary approaches were prespecified: (1) pattern-mixture models stratified by dropout timing, (2) multiple imputation under missing-not-at-random (MNAR) scenarios with δ-adjustment (δ = 0.2, 0.5 SD penalty favoring control), and (3) tipping-point analysis quantifying the magnitude of departure from MAR required to nullify the primary finding.

#### 2.6.5 Subgroup and covariate analyses

Treatment-effect heterogeneity was explored across four prespecified subgroups: baseline symptom severity (above/below median), tumor stage (I vs II–IIIA), age (<65 vs ≥65 years), and concurrent other therapy (yes/no). Interaction terms (treatment × subgroup) were incorporated into the MMRM; heterogeneity was tested via likelihood-ratio test at α = 0.10, consistent with lower power for interactions. These analyses are hypothesis-generating; multiplicity was not controlled. Separately, sensitivity models additionally adjusted for ECOG performance status and comorbidity burden to assess confounding robustness.

#### 2.6.6 Software and general considerations

Analyses were conducted in SAS 9.4 (PROC MIXED) and R 4.3 (lme4, mice packages). A comprehensive Statistical Analysis Plan (SAP), finalized and signed prior to database lock and unblinding, prespecified all endpoints, analysis populations, covariate handling, and inferential decision rules—including stopping boundaries if interim analyses were planned.

#### 2.6.7 Plan access

The statistical analysis plan is integrated into the study protocol and can be accessed upon publication.

### 2.7 Monitoring

#### 2.7.1 Data monitoring

A formal Data Monitoring Committee (DMC) was not convened. This decision reflects the intervention’s established safety profile—derived from prior trials (N > 500 cumulative exposure) showing no serious device-related events—and a primary endpoint of patient-reported symptoms rather than mortality or irreversible morbidity. An independent Contract Research Organization (CRO) assumed responsibility for ongoing quality oversight and regulatory compliance. Site monitoring followed a risk-based approach per ICH E6(R2): initiation visits prior to first enrollment, routine monitoring every 6–8 weeks (or after every 10 participants enrolled, whichever occurred first), and close-out visits within 30 days of last-participant-last-visit. Monitoring scope included verification of informed consent documentation, protocol adherence (with major/minor deviation classification), 100% source data verification for primary endpoints, adverse event ascertainment and coding accuracy, and investigational product accountability (dispensing logs, return reconciliation). Findings were documented in monitoring visit reports reviewed by the sponsor within 10 business days.

#### 2.7.2 Quality assurance

SDV followed a risk-based strategy: 100% verification was applied to primary symptom cluster scores, key secondary CPET parameters, and eligibility criteria; remaining eCRF fields underwent 20% random sampling per site. Discrepancies were classified as critical (affecting endpoint integrity), major, or minor, with resolution timelines of 3, 7, and 14 days, respectively.

All AEs were captured continuously via participant diaries and investigator assessment at each visit. Serious adverse events (SAEs) triggered expedited reporting: notification to the sponsor within 24 hours of investigator awareness, with complete narratives submitted within 5 calendar days. Events meeting regulatory criteria for Suspected Unexpected Serious Adverse Reactions (SUSARs) were reported to ethics committees and competent authorities within 7 days (fatal/life-threatening) or 15 days (other serious), per ICH E6(R2) and local regulations.

### 2.8 Monitoring

#### 2.8.1 Research ethics approval

Ethical Oversight The study adheres to the Declaration of Helsinki (2013 revision) and ICH E6(R2) Good Clinical Practice guidelines. Ethical approval was granted by the Institutional Ethics Committee of Beijing Hospital of Traditional Chinese Medicine, Capital Medical University (No. 2024BL02-122-05; date:10/14/2025). Protocol amendments—whether safety-related, administrative, or substantive—require prospective ethics committee approval; participating sites will be notified within 7 days of amendment authorization, with re-consent obtained if amendments materially alter the risk–benefit profile.

#### 2.8.2 Informed consent

Written informed consent is obtained before enrollment and any study-specific procedures. Trained physician-investigators conduct the consent discussion, covering: study rationale and design, visit schedule and procedures, foreseeable risks (graded by likelihood and severity), anticipated benefits (individual and societal), available alternatives, voluntariness of participation, and the unconditional right to withdraw without impact on standard care.

Participants receive ≥24 hours to deliberate before signing; questions may be addressed in person or by telephone. Each participant retains a signed copy; the original is filed in the investigator site file. For individuals with limited literacy, an impartial witness—independent of the study team—attests that all information was accurately conveyed and consent was given voluntarily; both the participant’s mark and the witness’s signature are documented.

Participants who experience AEs or SAEs judged by the investigator to be possibly, probably, or definitely related to trial procedures will receive prompt medical care at no cost.

#### 2.8.3 Additional consent provisions

Consent for the collection, storage, and use of biological specimens—including assays of HPA axis hormones (cortisol, ACTH), inflammatory cytokines (IL-6, TNF-α), and gonadal hormones (estradiol, progesterone)—is incorporated within the main informed consent form.

#### 2.8.4 Confidentiality and data protection

Participant identities are protected through pseudonymization: unique study codes replace personal identifiers on all case report forms and datasets. The linkage key is stored separately, accessible only to the site principal investigator and designated data manager.

## 3 Discussion

Symptom-cluster research in oncology has expanded over the past decade, yet most work remains descriptive. Studies have concentrated on cataloging how different anticancer regimens—chemotherapy, radiotherapy, endocrine therapy—shape cluster composition, and on cross-sectional surveys of symptom prevalence (34, 35). The existing literature predominantly comprises retrospective and observational studies that have provided preliminary insights into the incidence of symptom clusters and patterns of symptom co-occurrence (36). This evidence base has nonetheless delineated consistent patterns. The fatigue-centered cluster, including pain, dyspnea, etc., which emerges across cohorts as the most prevalent and the most functionally impairing, correlating with reduced return-to-work rates and diminished HRQoL scores (26, 27, 37, 38).

The clinical burden of fatigue-centered clusters extends beyond daily function (19, 39). Because fatigue, dyspnea, and pain are inherently subjective and captured primarily through patient self-report, they are often under-recognized in routine oncology practice. Management options remain limited. A meta-analysis of 113 randomized trials found that interventions lasting a mean of 14 weeks produced modest improvements in cancer-related fatigue, with weighted effect sizes of 0.30 for exercise, 0.27 for psychological interventions, and 0.26 for their combination, compared with 0.09 for pharmacological interventions (33). Consistent with these findings, the 2024 ASCO–SIO guideline recommends exercise, cognitive-behavioral therapy, and mindfulness-based programs for cancer survivors, while advising against the routine use of psychostimulants, antidepressants, or wakefulness agents for cancer-related fatigue (25). Nevertheless, these recommendations primarily address fatigue as an individual symptom, and evidence regarding interventions targeting fatigue-centered symptom clusters remains limited.

Shenqi Shiyiwei Granule is a Chinese patent medicine formulated under the guidance of traditional Chinese medicine theory. Multiple clinical trials have demonstrated its efficacy in ameliorating fatigue and related symptoms, and it has been recommended by several Chinese clinical guidelines and expert consensus statements for the treatment of disease-induced fatigue. The present study aims to enroll patients with well-defined pathological subtypes and treatment stages who present with the fatigue-centered symptom cluster. Through rigorous study design and implementation, this trial seeks to determine whether the investigational drug can effectively improve the fatigue-centered symptom cluster, thereby providing high-quality evidence for the management of fatigue-centered symptoms in breast cancer survivors. Furthermore, through exploratory biomarker assessments—including hypothalamic-pituitary-adrenal axis hormones, cytokine profiling, and gonadal hormone measurements—this study endeavors to elucidate potential therapeutic targets and the molecular mechanisms underlying the interrelationships among symptoms within the cluster, thereby establishing a foundation for future mechanistic and interventional research.
