# Supplementary material for: Shenqi Shiyiwei Granule for breast cancer related fatigue-centered symptom cluster: protocol of a multicenter randomized double-blind placebo-controlled trial
Source: Front Oncol. 2026 Oct 19;16:1941699. doi: 10.3389/fonc.2026.1941699 (PMC13724569; doi:10.3389/fonc.2026.1941699)
Supplement: Supplementary file 1 [file Table1.docx]

| Item | Details |
| --- | --- |
| Herbal components | Ren Shen (Ginseng Radix et Rhizoma); Huang Qi (Astragali Radix); Dang Gui (Angelicae Sinensis Radix); Tian Ma (Gastrodiae Rhizoma); Shu Di Huang (Rehmanniae Radix Praeparata); Ze Xie (Alismatis Rhizoma); Jue Ming Zi (Cassiae Semen); Lu Jiao (Cervi Cornu); Tu Si Zi (Cuscutae Semen); Xi Xin (Asari Radix et Rhizoma); and Gou Qi Zi (Lycii Fructus) |
| Excipients | Dextrin and steviol glycosides |
| Unit strength | 2 g per sachet, equivalent to 5 g of herbal materials |
| Manufacturer | Jiangxi Shangao Pharmaceutical Co., Ltd., China |
| Regulatory information | NMPA approval number Z10980002 |
| Quality standard | Chinese Pharmacopoeia, 2025 edition, Part I, and the approved product standard (Notice No. 2026B01304) |
| Administration | One sachet orally three times daily for 60 days |

**Table S1** Composition and product information of Shenqi Shiyiwei Granule
